# Management of Known Cushing’s Disease in a Nonsurgical Candidate Secondary to a History of Hemorrhagic Stroke Case Report

**DOI:** 10.1155/crie/2844939

**Published:** 2026-02-06

**Authors:** Kyle Distler, Jean Ramos Cardona, Suzanne Martinez

**Affiliations:** ^1^ Internal Medicine Residency Program, HCA Florida Orange Park Hospital, 2001 Kinsgely Avenue, Orange Park, 32073, Florida, USA

**Keywords:** case report, Cushing’s disease, hemorrhagic stroke, medical management of Cushing’s disease, surgical management of pituitary macroadenoma

## Abstract

**Background:**

Cushing’s disease can present with hyperglycemia, hypertension, electrolyte abnormalities, headaches, confusion, gastrointestinal (GI) bleeds, and more. Macroadenomas of the pituitary causing cortisol excess can complicate these cases of patients with a recent hemorrhagic stroke with the medical complexities found within both disease processes of hemorrhagic stroke and Cushing’s disease.

**Case:**

This is a 61‐year‐old female patient who returned from a rehabilitation facility after confusion, abdominal pain, vaginal bleeding, and weakness. History included hypertension, hypothyroidism, type 2 diabetes mellitus, suspected Cushing’s disease, hemorrhagic stroke, and a lumbar compression fracture. Blood pressure was 195/87 with a potassium of 2.0. X‐ray showed a nonobstructive bowel gas pattern, and computed tomography (CT) of the abdomen and pelvis was concerning for stercolitis, multiple pancreatic cysts, and atelectasis. Insulin, intravenous (IV) fluids, and electrolyte replacement were initiated. She developed a deep venous thrombosis (DVT) in the right lower extremity and was placed on enoxaparin. Worsening of GI bleeding occurred, and an inferior vena cava filter was placed. Osilodrostat was started. Colonoscopy showed ulcerations in the sigmoid colon. Pathology showed no findings concerning dysplasia or malignancy. Osilodrostat was increased to 2 mg twice a day. She was discharged home, with follow‐ups for resection of her macroadenoma, biopsy of uterine endometrium, and genetic testing.

**Discussion/Conclusions:**

The clinical manifestations found in this case are largely due to hypercortisolism, and while she is going to still have additional testing including biopsy of the fibroid, colorectal surgical evaluation for hemorrhoids, and genetic testing with confirmatory lab work per endocrinology outpatient, her illness was medically uncontrolled. As osilodrostat takes a couple weeks to a couple months for full control with frequent cortisol checks, adjustments including insulin, blood pressure control, electrolyte corrections, and more should be considered.

## 1. Background

Cushing’s disease, a form of Cushing’s syndrome caused by an adrenocorticotropic hormone (ACTH)–secreting pituitary adenoma, presents with a complex constellation of clinical features including hyperglycemia, hypertension, electrolyte abnormalities, visual disturbances, cognitive changes, proximal muscle weakness, osteoporosis, gastrointestinal (GI) bleeds, and much more [1, 2]. In patients who have experienced a hemorrhagic stroke within the last 6 months, an ACTH‐secreting macroadenoma of the pituitary gland with increased subsequent cortisol production can further complicate recovery.

Surgical resection is considered the first‐line therapy in individuals with Cushing’s disease with pituitary, ACTH‐producing, macroadenoma. However, one‐third of those receiving this surgery do not achieve remission and require additional medical treatment [1, 2]. Diagnosis can be achieved with two of three of the following abnormal: late night salivary cortisol, overnight 1‐mg dexamethasone suppression test, and low‐dose 2‐day dexamethasone test [1]. Medical management of this disease includes medications that target glucocorticoid receptors, pituitary‐directed agents, and adrenal‐directed drugs to attempt to maintain control of this axis in those who cannot be controlled via procedure [1]. The recommended surgical approach is one with a combined team of an otolaryngologist and a neurosurgeon [2]. Current guidelines recommended delaying elective neurosurgical interventions for at least 6 months following a hemorrhagic stroke [3, 4]. We present the case of a patient with a preexisting initial workup outpatient concerning for Cushing’s disease who presented with confusion, hypokalemia, weakness, persistent vaginal bleeding, and GI bleeding, 3 months after a hemorrhagic stroke.

## 2. Case

A 61‐year‐old woman with a history of hypertension, hypothyroidism, type 2 diabetes mellitus, hemorrhagic stroke (3 months prior), and lumbar compression fracture was readmitted from a rehabilitation facility due to confusion, abdominal pain, vaginal bleeding, and a potassium of 2.0 mmol/L. In the previous month, outpatient workup showed initial findings concerning Cushing’s disease with recommended ongoing evaluation and lab work. Initial blood pressure in the Emergency Department was 195/87 mmHg. Uncontrolled hyperglycemia (glucose 332 mg/dL) and a bicarbonate level above 40 mmol/L were present with computed tomography (CT) scan of the abdomen and pelvis showing findings concerning for stercoral colitis, persistent fluid within the endometrial canal, multiple small pancreatic cysts concerning for intraductal papillary mucinous neoplasms, adrenal enlargement, and bibasilar atelectasis.

**Figure 1:**
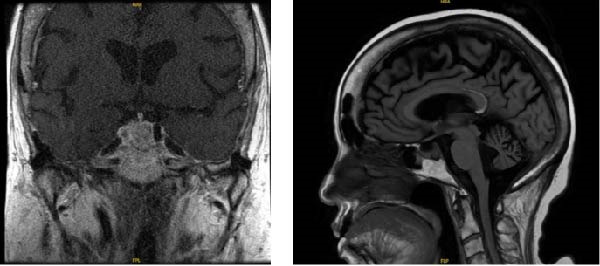
Pituitary MRI shows a 1.3 cm hypoenhancing nodule in the posterior aspect of the sella suggestive of a macroadenoma.

Initial management included intravenous (IV) fluids and electrolyte repletion with initiation of insulin therapy. In the Emergency Department, Gastroenterology started mineral oil and polyethylene glycol, which successfully resulted in multiple large bowel movements and improvement in CT findings. Endocrinology was consulted due to her complex history of suspicion for Cushing’s disease with ongoing outpatient workup about a month prior. Thyroid stimulating hormone (TSH) was suppressed at 0.34 µU/mL, with a free thyroxine of 1.04 ng/dL, and her levothyroxine was increased to 100 mg/day, which was because previous admission had shown TSH of 0.29 mcUnt/mL with a free T4 of 0.72 ng/dL with clinical and laboratory confirmation of central hypothyroidism. During the time of her outpatient workup for Cushing’s disease earlier that month, prolactin was mildly elevated at 26.6 ng/mL, with the 1 mg dexamethasone suppression test showing a cortisol of 40.8 mcg/dL and an ACTH of 75.3 pg/mL. This was consistent with previous workup the month prior at another hospital where the results were a cortisol level of 43.7 mcg/dL and ACTH of 186 pg/mL. Follicle‐stimulating hormone (FSH) was less than 0.7, and luteinizing hormone (LH) was less than 0.2 mIU/mL at that time. Estradiol was 48.4 pg/mL not on hormone replacement therapy, and insulin‐like growth factor‐1 (IGF‐1) was low at 37 ng/mL. CT of abdomen and pelvis at that time showed enlarged adrenal glands bilaterally. Further laboratory evaluation and pituitary magnetic resonance imaging (MRI) findings are summarized in Table 1 and Figure 1. The patient was recommended to follow up with neurosurgery and Otolaryngology for surgical removal of pituitary macroadenoma. However, she was at rehab after previous hospital admission from weakness and falls, and given that her hemorrhagic stroke was within the last 4 months, she was not a current surgical candidate and was recommended to be managed medically at that time.

**Table 1:** Endocrinology laboratory test.

Test	Result	Reference
ACTH	75.3	7.2–63.3 pg/mL
Cortisol	40.80	AM: 4.3–22.40 mcg/dL
Prolactin	26.7	2.1–17.7 ng/mL
LH	<0.2	0–200 mInU/mL
FSH	<0.7	0–200 mInU/mL
TSH	0.34	0.55–4.7 mcUnt/mL
Free T4	1.04	0.89–1.76 ng/dL
A1C	7.2	<5.7

Electrolyte and blood pressure lability necessitated the consultation of nephrology on day 5, who started hydrochlorothiazide, continued current blood pressure regimen, and started 5% dextrose in water (D5W) at 50 cc/h. On day 7 of this hospitalization, the patient developed a right lower extremity deep venous thrombosis (DVT) and was started on subcutaneous enoxaparin. Upper and lower endoscopies were performed on day 8 given persistent GI bleed and revealed ulcerations in the sigmoid colon and a polypoid lesion at the ileocecal valve.

To address the patient’s hypercortisolemia, osilodrostat was initiated and titrated up to 2 mg twice daily. Milligrams twice a day 20. Cortisol was to be repeated outpatient on follow‐up with endocrinology. This target therapy was used to help manage this patient’s cortisol‐driven complications, including electrolytes disturbances, hyperglycemia, bone loss leading to a lumbar compression fracture, and hypertension. Given persistent GI bleeding in the setting of anticoagulation for a DVT, the patient was recommended for inferior vena cava filter which was eventually placed on day 13. The patient continued high‐dose pantoprazole for GI protection and was to follow up with Gastroenterology outpatient.

This individual was discharged home with home health support and outpatient follow‐ups for otolaryngology and neurosurgery for a combined approach for resection of her cortisol‐producing macroadenoma. She was also followed up with Endocrinology for continued workup with genetic testing for possible multiple endocrine neoplasia (MEN)–related syndromes as well as additional supportive evidence to confirm Cushing’s disease with measures such as late‐night salivary cortisol and 24‐h urinary cortisol to be completed after discharge. Lastly, she was to see Obstetrics and Gynecology on discharge for endometrial biopsy and abnormal vaginal bleeding management.

## 3. Discussion

This case highlights the multisystem effects of severe hypercortisolism in an individual who was not eligible for surgical resection at that time given her recent history of a hemorrhagic stroke. The patient showed classic and severe manifestation of excess cortisol including GI erosions, uncontrolled elevated blood pressure and severe hypokalemia despite medications, profound proximal muscle weakness leading to an injurious fall, vision disturbances secondary to hyperglycemia and mass‐producing lesion on the anterior pituitary affecting the optic nerve, hypercoagulability resulting in a DVT requiring an inferior vena cava filter, altered mentation, and vaginal bleeding (previously diagnosed on prior admission with uterine fibroid) [1, 2].

These complications demonstrate the systemic burden of uncontrolled Cushing’s disease, particularly in patients in whom surgery must be delayed. In this case, the patient continues to undergo further evaluation, including biopsy of the uterine fibroid, colorectal surgical assessment for GI bleeding, and genetic testing to rule out syndromic causes such as multiple endocrine neoplasia type 1 (MEN1). In her case with concern for mucinous neoplasms in the pancreas and pituitary tumor, ruling out MEN1 is necessary. To note, however, her calcium was within normal limits as was her parathyroid hormone (PTH) while inpatient. Medical therapy with osilodrostat, while effective, requires frequent cortisol level monitoring to assess efficacy and avoid adrenal insufficiency [1, 2]. However, this patient had contraindications to utilizing other cortisol‐lowering therapy such as mifepristone due to abnormal vaginal bleeding [5, 6]. Given that this patient had increased collection of endometrial fluid, thickened endometrial stripe, and abnormal vaginal bleeding, medications blocking progesterone receptors were unable to be used in her case [5]. Clinical response may take weeks to months with supportive care being essential [7]. Osilodrostat’s blockage of 11‐beta hydroxylase was safer to utilize in her case as mifepristone’s blocking of glucocorticoid receptors could potentiate worsening of vaginal bleeding, increasing in wall thickness, and shedding of the endometrium [6].

Nonsurgical treatment of Cushing’s disease includes many strategies. It is important to note that presurgical medical therapy with cortisol‐lowering or receptor‐blocking agents is standard in many patients to improve metabolic status and reduce surgical risk. Although optimal treatment in cases like these is surgical transsphenoidal resection of the pituitary mass, medical management is often required when surgery is delayed for any reason such as seen here in our patient’s case of recent hemorrhagic stroke. Medical treatment options include adrenal steroidogenesis inhibitors (e.g., ketoconazole, osilodrostat, and metyrapone), corticotroph agents (e.g., cabergoline), and glucocorticoid receptor antagonist (e.g., mifepristone) [1]. Additionally, bilateral adrenalectomy can provide definitive cure with daily glucocorticoid and mineralocorticoid treatment for life, but this is reserved for refractory or recurrent disease cases after unsuccessful macroadenoma surgery or failure of medical management [1].

## 4. Conclusions

This patient’s difficult situation of hemorrhagic stroke within 3 months and severe Cushing’s disease with macroadenoma led to disease manifestations of classic GI bleeding, vaginal bleeding, weakness and falls, electrolyte and blood pressure derangements, and hypercoagulability. While ultimately controlled with insulin, electrolyte and blood pressure control, osilodrostat, and IV fluids, this patient suffered significant morbidity due to delayed control. This case report serves as a review of complications of Cushing’s disease in someone with recent hemorrhagic stroke, GI, and vaginal bleeding.

## Author Contributions

Kyle Distler is the lead author, editor, cared for the patient in the hospital, and submitted the manuscript—kyle.distler@hcahealthcare.com. Jean Ramos Cardona edited the manuscript and aided in the creation of table/figures—jean.ramoscardona@hcahealthcare.com. Suzanne Martinez is the primary faculty on case report and editing and final approvals of the manuscript—suzanne.martinez@hcahealthcare.com.

## Funding

No funding sources have been utilized for this manuscript.

## Disclosure

The views expressed in this publication represent those of the author(s) and do not necessarily represent the official views of HCA Healthcare or any of its affiliated entities. All authors have read and approved the final version of the manuscript.

## Ethics Statement

Ethical approval to report this case was obtained from GME PubClear 2025 (MS #2553) after obtaining a signed consent form from the patient and having this approved by this IRB. GME PubClear is a process used to approve HCA Healthcare publications for external dissemination.

## Consent

A written informed consent was obtained from the patient for publication of this case report and any accompanying images. Production of this manuscript and consented by the patient with a signed consent form by this individual and is stored by the primary author, Kyle Distler.

## Conflicts of Interest

HCA employs these authors.

## Data Availability

Kyle Distler had full access to all of the data in this study and takes complete responsibility for the integrity of the data and the accuracy of the data analysis.
